# Isolated systolic hypertension and its associated risk factors in Iranian middle age and older population: a population-based study

**DOI:** 10.1186/s12872-022-02856-7

**Published:** 2022-09-27

**Authors:** Ali Hosseinzadeh, Hossein Ebrahimi, Ahmad Khosravi, Mohammad Hassan Emamian, Hassan Hashemi, Akbar Fotouhi

**Affiliations:** 1 Department of Epidemiology, School of Public Health, Shahroud University of Medical Sciences, Shahroud, Iran; 2 Randomized Controlled Trial Research Center, Shahroud University of Medical Sciences, Shahroud, Iran; 3 Ophthalmic Epidemiology Research Center, Shahroud University of Medical Sciences, Shahroud, Iran; 4 Noor Ophthalmology Research Center, Noor Eye Hospital, Tehran, Iran; 5 Department of Epidemiology and Biostatistics, School of Public Health, Tehran University of Medical Sciences, PO Box: 14155-6446, Tehran, Iran

**Keywords:** Isolated systolic hypertension, Older adult, Risk factor, Prevalence, Iran

## Abstract

**Background:**

Isolated systolic hypertension (ISH), is the most common form of hypertension in older adults. However, the ISH prevalence is not well known in many developing countries such as Iran. This study was conducted to determine the ISH prevalence and its related risk factors in an Iranian population.

**Methods:**

Data were obtained from the second phase of the Shahroud eye cohort study (ShECS) in 2014. ShECS is a longitudinal population-based study which the first phase had been conducted in 2009 using the stratified multistage cluster sampling design on 5190 people aged 40 to 70 years. The ISH prevalence was determined based on the eighth Joint National Commission guidelines for different demographic variables. The associated risk factors were estimated by multiple logistic regression and a two-tailed p-value less than 0.05 was considered significant.

**Results:**

The ISH prevalence was 15.89% (95% CI: 14.88–16.96). It was 15.68% (14.12–17.39) and 15.87% (14.54–17.29) for men and women, respectively. The prevalence of ISH increased significantly with increasing age. The 65–70 compared to 45–69 year age group (OR = 4.21), body mass index (OR = 1.03), diabetes (OR = 1.64), retirement, compared to practitioner job (OR = 1.53), and waist to hip ratio (WHR) (OR = 9.81) were significantly associated with ISH prevalence.

**Conclusions:**

ISH is highly prevalent among the older adult population in Iran. Given the risk of cardiovascular disease associated with ISH, it is recommended to conduct education and public health interventions to improve the detection, prevention, and treatment of ISH.

## Introduction

Hypertension is a serious medical condition and a major cause of premature death especially in low- and middle-income countries [1]. The disease is estimated cause to 10.7 million deaths (about 33.2% of deaths attributed to all risk factors) and 212 million Disability Adjusted Life Years (DALYs) (about 20.9% of DALYs attributed to all risk factors) worldwide [2]. According to the result of the global burden of disease study, hypertension is directly responsible for about 45% of deaths due to heart disease and 51% of deaths due to stroke [3]. Given the increase in the world's elderly population, the prevalence of this disease is expected to increase to 31.9% by 2025 [4, 5]. Hypertension has many different forms, including systolic-diastolic hypertension (SDH), isolated systolic hypertension (ISH), and isolated diastolic hypertension (IDH) [6, 7]. The ISH, which is defined as systolic blood pressure ≥ 140 mmHg and diastolic blood pressure < 90 mmHg [8], is the most common form of hypertension in older adults and affects approximately 50% of people over 60 years of age [9]. However, in recent decades the ISH prevalence has also increased in young adults due to the epidemic of overweight and obesity [10, 11]. The prevalence of ISH has been reported different from 6.51 to 35%, which depend on the definition of ISH and distribution of age, sex, race and other variables in the studied populations [9, 12–15]. It has shown that systolic blood pressure plays a greater role in cardiovascular disease than diastolic blood pressure [16]. Also, other epidemiological and interventional studies have shown that ISH considerably increased the risk of cardiovascular disease, stroke, chronic kidney disease, and dementia [17–22]. However, the prevalence and incidence of ISH in many developing countries, such as Iran, are not well known and this form of hypertension has received less attention as a strong risk factor. Therefore, the present study aimed at determining the prevalence of ISH and its related risk factors in the Iranian population.

## Methods

The present study was conducted based on the data obtained from the second phase of the Shahroud eye cohort study (ShECS) in 2014. Shahroud eye cohort study is a longitudinal population-based study. The first phase of this study was conducted in 2009 in people aged 40–64 years old in Shahroud, northeast Iran. In ShECS, using the stratified multistage clustered sampling design, a total of 6311 people were randomly selected. For this purpose, each health care center (Shahroud has 9 comprehensive health service centers) was used as a stratum in Shahroud, and in each stratum were determined the number of clusters based on the population covered. The number of 300 clusters, and at least 20 individuals aged 40 to 64 years were selected from each cluster to participate in the study [23]. Of the 6311 individuals selected for this study, 5190 people (82.23%) participated in the study. After obtaining informed consent from the participants, in addition to demographic information including sex, age, economic status, education years, employment status, marital status, and smoking, participants underwent clinical examination, anthropometric examinations, blood pressure measurements, optometry and ophthalmology examinations, and tests. Of all 5190 people in the first phase, 453 people (8.7%) did not participate in the second phase of the study and 103 people had missing data. Finally, 4634 people were analyzed in this study. The study protocol was reviewed and approved by the Ethics Committee of Shahroud University of Medical Sciences. The details of the methodology of this study have already been published [23].

Blood pressure was measured by trained nursing staff twice for each participant using an Omron M3 Intellisense (HEM-7051-E, Omron Corp, Tokyo, Japan) automated electronic oscillometric blood pressure monitor, with medium/large cuffs at the right arm in the sitting position. The final blood pressure was the mean of the two measurements.

In the present study, consistent with the International Society of Hypertension guidelines [8], SDH was defined as systolic BP ≥ 140 mmHg and diastolic BP ≥ 90 mmHg, and ISH was defined as systolic BP ≥ 140 mmHg and diastolic BP < 90 mmHg. The prevalence of ISH was estimated in different age groups, sex, marital status, job status, diabetes status, abdominal obesity (waist circumference ≥ 102 cm in men and ≥ 88 cm in women), education years, and economic status with 95% confidence intervals. In ShECS, the economic status has been determined through principal component analysis on home assets [24].

The association between the categorical variables was assessed using the chi-square test. For quantitative variables, means were compared using the independent t-test. Variables that had a P-value of less than 0.2 in univariate analysis were included in multivariate analyses. The associated risk factors were estimated by multiple logistic regression analysis with the presence of ISH as the dependent variable. A two-tailed P-value less than 0.05 was considered significant. The effects of cluster sampling were considered in calculating the standard errors and confidence intervals.

## Results

Among the 4737 participants in the second phase of the ShECS, the data required for this study were available for 4634 (41.13% were male) and analyzed for this report. The range and mean age of participants were 45–69 and 55.85 ± 6.21 years respectively. Most of the participants were married (89.73%) and in a low economic class (56.44%).

The mean education years, fasting blood sugar (FBS), and BMI of participants were 7.29 ± 4.66 years, 107.16 ± 40.52 mg/dl, and 28.87 ± 4.99 kg/m^2^, respectively. Other baseline characteristics of participants are provided in Table 1 according to isolated systolic hypertension.

**Table 1:** Baseline characteristics of participants with and without Isolated Systolic Hypertension

Variables	With ISH	Without ISH	Total	*P*-value
	N (%),	N (%),	N (%),	
	Mean ± SD	Mean ± SD	Mean ± SD	
Age Groups (Year)				
45–49	58 (6.78)	797 (93.22)	855 (18.45)	< 0.001
50–54	149 (11.85)	1108 (88.15)	1257 (27.15)	
55–59	184 (15.85)	977 (84.15)	1161 (25.05)	
60–64	183 (21.73)	659 (78.27)	842 (18.17)	
65–69	158 (30.51)	361 (69.56)	519 (11.18)	
Gender				
Men	299 (15.69)	1607 (84.31)	1906 (41.13)	0.862
Women	433 (15.87)	2295 (84.13)	2728 (58.87)	
Educational level				
Illiterate	117 (22.81)	396 (77.19)	513 (11.08)	< 0.001
Primary	241 (16.45)	1224 (83.55)	1465 (31.63)	
Guidance	113 (16.24)	583 (83.76)	696 (15.03)	
Highschool	195 (13.58)	1241 (86.42)	1436 (31.01)	
College	66 (12.67)	455 (87.33)	512 (11.25)	
Economic Status				
Low	287 (18.76)	1273 (81.03)	1555 (33.56)	< 0.001
Moderate	238 (15.36)	1331 (84.67)	1549 (33.43)	
High	207 (13.31)	1360 (86.51)	1530 (33.02)	
Marital status				
No	82 (17.23)	394 (82.77)	476 (10.27)	0.361
Yes	650 (15.63)	3508 (84.37)	4158 (89.73)	
Job				
Practitioner	90 (9.83)	826 (90.17)	916 (19.77)	< 0.001
Retired	249 (19.78)	1010 (80.22)	1259 (27.17)	
Housekeeper	385 (16.11)	2005 (83.39)	2390 (51.58)	
Others	8 (11.59)	61 (88.41)	69 (1.49)	
Current smoking				
No	639 (16.09)	3332 (83.91)	3971 (85.69)	0.174
Yes	93 (14.03)	570 (85.97)	663 (14.31)	
Yes	16 (26.23)	45 (73.77)	61 (1.32)	
Diabetes				
Nondiabetic	466 (13.31)	3033 (86.68)	3499 (76.15)	< 0.001
Diabetic	259 (23.63)	837 (76.37)	1096 (23.85)	
Abdominal obesity				
No	187 (13.36)	1213 (86.64)	1400 (30.25)	< 0.003
Yes	544 (16.85)	2684 (83.15)	3228 (69.75)	
Education (Year)	6.41 ± 4.73	7.47 ± 4.63	7.31 ± 4.66	< 0.001
Body Mass Index (kg/m^2^)	29.67 ± 4.92	28.73 ± 4.99	28.88 ± 4.99	< 0.001
Fasting Blood Sugar (mg/dL)	116.91 ± 47.30	105.34 ± 38.86	107.16 ± 40.52	< 0.001
Triglyceride (mg/dL)	192.11 ± 106.77	173.72 ± 97.08	176.62 ± 98.88	< 0.001
Total cholesterol (mg/dL)	198.52 ± 43.22	197.77 ± 42.26	197.92 ± 41.57	0.563
Glycated haemoglobin (%)	5.81 ± 1.52	5.33 ± 1.41	5.41 ± 1.44	< 0.001
Height (cm)	158.87 ± 8.86	159.71 ± 8.66	159.57 ± 8.68	< 0.01
Weight (kg)	74.73 ± 12.56	73.09 ± 12.71	73.35 ± 12.69	< 0.001
Waist circumference (cm)	104.51 ± 10.58	101.06 ± 10.83	101.61 ± 10.86	< 0.001
Wrist circumference (cm)	17.22 ± 1.36	17.07 ± 1.33	17.09 ± 1.34	< 0.005
Hip circumference (cm)	106.05 ± 9.74	104.96 ± 9.26	105.13 ± 9.34	< 0.004
Waist to Hip Ratio	0.99 ± 0.07	0.96 ± 0.08	0.96 ± 0.7	< 0.001

*ISH* isolated systolic hypertension, *SD* Standard Deviation

**Fig. 1:**
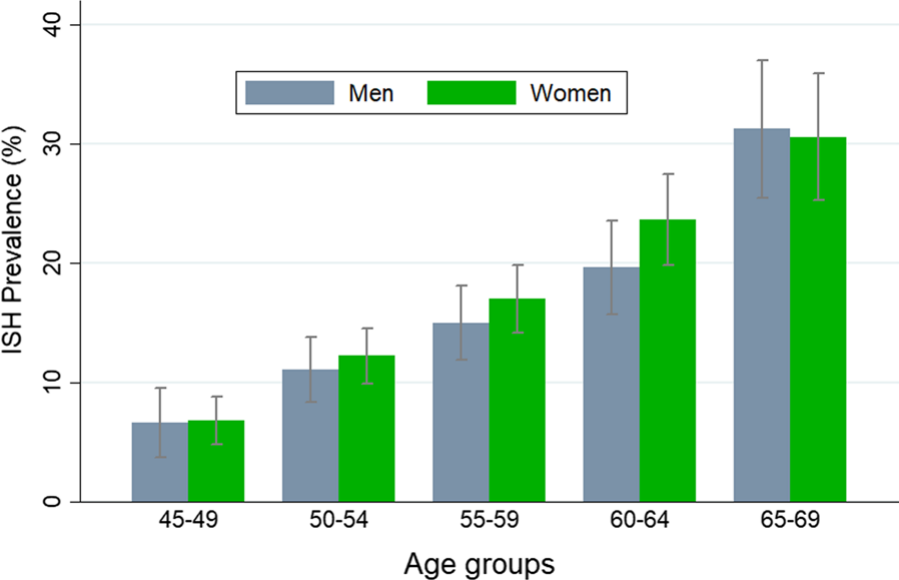
The prevalence of isolated systolic hypertension (ISH) in the study population by age and gender. Error bars indicate 95% confidence intervals for prevalence

The overall prevalence of ISH was 15.89% (95% CI: 14.88–16.96%). The prevalence of ISH increased significantly with increasing age and it was 30.51% (26.68–34.60%) in the 65–70 years age group (Table 2). In all age groups, the ISH prevalence was higher in women than men, except in the age group of 65 to 70 years (Fig. 1), although this difference was not statistically significant.

**Table 2:** Prevalence of isolated systolic hypertension by sociodemographic variables in the study population

Variables	Prevalence of ISH	95% Confidence interval		*P*-value
		Lower	Upper	
Age groups (year)				
45–49	6.78	5.27	8.67	< 0.001
50–54	11.85	10.17	13.75	
55–59	15.85	13.85	18.06	
60–64	21.73	19.07	24.65	
65–69	30.51	26.68	34.60	
Gender				0.861
Men	15.68	14.12	17.39	
Women	15.87	14.54	17.29	
Education level				< 0.001
Illiterate	22.81	19.37	26.64	
Primary	16.45	14.63	18.43	
Guidance	16.23	13.67	19.16	
Highschool	13.57	11.91	15.45	
College	12.66	10.07	15.81	
Economic status				< 0.001
Low	18.75	16.87	20.79	
Middle	15.36	13.65	17.24	
High	13.31	11.71	15.09	
Marital status				0.362
Yes	15.63	14.55	16.76	
No	17.22	14.08	20.89	
Job				< 0.001
Practitioner	9.82	8.05	11.93	
Retired	19.77	17.66	22.07	
Housekeeper	16.11	14.68	17.63	
Others	11.59	5.87	21.61	
Current smoking				0.174
Yes	14.02	11.58	16.88	
No	16.09	14.98	17.26	
Diabetes				< 0.001
Diabetic	23.63	21.21	26.23	
Nondiabetic	13.31	12.23	14.48	
Body Mass Index				< 0.001
< 18.5	4.34	1.07	16.01	
18.5–24.9	12.84	10.82	15.17	
25–29.9	15.43	13.89	17.11	
≥ 30	18.05	16.31	19.93	
Abdominal obesity				< 0.003
Yes	16.85	15.59	18.18	
No	13.35	11.67	15.24	

*ISH* Isolated Systolic Hypertension, *CI* Confidence Interval

The prevalence of ISH was higher in the low economic class 18.96% (95% CI: 17.10–20.98%). The prevalence of ISH significantly decreased with increasing education level and it was 22.81 (95% CI: 19.37–26.64) and 12.66 (95% CI: 10.07–15.81) in illiterate and college-educated respectively (Table 2).

On forward stepwise multiple logistic regression analysis, 65–70 years age group [OR = 4.21 (3.01–5.88)], BMI [OR = 1.03 (1.01–1.05)], diabetes [OR = 1.64 (1.36–1.98)], retirement [OR = 1.53 (1.17–2.01)], and waist to hip ratio (WHR) [OR = 9.81 (3.14–30.61)] were significantly associated with higher odds of ISH (Table 3).

**Table 3:** Simple and multiple logistic regression models for the association of various risk factors with isolated systolic hypertension

Independent variables	Crude Odds Ratio	*P*-value	Adjusted Odds Ratio	*P*-value
	(95% CI)		(95% CI)	
Age groups (year)				
45–49	Reference	–	Reference	–
50–54	1.84 (1.35–2.51)	< 0.001	1.68 (1.23–2.51)	< 0.001
55–59	2.58 (1.85–3.61)	< 0.001	2.12 (1.51–2.98)	< 0.001
60–69	3.81 (2.79–5.21)	< 0.001	2.91 (2.08–4.06)	< 0.001
70–74	6.03 (4.41–8.23)	< 0.001	4.21 (3.01–5.88)	< 0.001
Education (year)	0.95 (0.93–0.96)	< 0.001	0.98 (0.96–1.00)	0.113
Economic status				
High	Reference	–	Reference	–
Middle	1.51 (1.24–1.82)	< 0.001	1.05 (0.84–1.31)	0.632
Low	1.18 (0.96–1.44)	0.112	0.99 (0.81–1.22)	0.991
Job type				
Practitioner	Reference	–	Reference	–
Retried	2.26 (1.77–2.87)	< 0.001	1.53 (1.17–2.01)	0.002
Housekeeper	1.76 (1.40–2.21)	< 0.001	1.21 (0.93–1.57)	0.146
Others	1.20 (0.57–2.51)	0.63	0.97 (0.48–1.96)	0.948
Diabetes	2.01 (1.69–2.41)	< 0.001	1.64 (1.36–1.98)	< 0.001
Current smoking	0.85 (0.67–1.08)	0.170	0.91 (0.69–1.19)	0.374
Body Mass Index	1.04 (1.02–1.05)	< 0.001	1.03 (1.01–1.05)	0.002
Waist to Hip Ratio	47.32 (16.79–133.36)	< 0.001	9.81 (3.14–30.61)	0.001

*CI* Confidence Intervals

## Discussion

In this study, ISH prevalence for the overall population, men, and women was 15.89%, 15.68%, and 15.87% respectively. To the best of our knowledge, no reliable study has previously investigated the ISH prevalence in Iran. Therefore, a valid comparison is not possible. Only in one study, the ISH incidence was reported to be 5.7 / 1000 people person-years in Iran [25]. The results of previous studies of ISH prevalence in different parts of the world are also sparse. However, the estimated prevalence of ISH in the current study appears to be slightly higher than the reported ISH prevalence in other countries. For example, in Taiwan, the prevalence of ISH has been reported as 12.6% in the overall population, 12.3% in males, and 12.9% in females [14]. The prevalence of ISH has been reported 7.6% in China and 10.1% in South Korea and 35% in Portugal [9, 13, 26] and 10.2% among the Mongolian population [15]. In western countries, the prevalence of ISH has been reported at 9.4% in the USA, and 8.1% in Canada [11, 27].

One of the most important factors that may cause the differences in the results of various studies is the age distribution of the study population [5, 27, 28]. The findings of the present study showed that the ISH prevalence increased with age increasing. So that the ISH prevalence in the 65 to 70 years age group was about 4.5-fold higher than in the 45–49.9 years age group. These findings are in line with the results of Framingham study. In the Framingham heart study prevalence of ISH has been reported 35% to 40% in the age group of 50–59 years and 65% to 70% in the age group of over 60 years [29]. In the study conducted in the USA, the ISH prevalence has been reported at 6% in the age group of 40–59 years, and 29.6% in the age group of over 60 years [11]. In Korean adults, also, the prevalence of ISH has been reported 20.2% in the age group of 40—49 and 63.2%, in the age group over 70 years [9].The studies conducted in the USA [30], South Korea [31], and China [26] also have been shown that the ISH prevalence increased steadily with increasing age [28]. Previous studies have shown that with increasing age, arteries tend to lose their elasticity with reduced compliance to the stroke volume [31–33]. Therefore, the increases in ISH prevalence with age may be in relationship to arteriosclerosis and an age-dependent decrease in compliance of the aorta and large capacitance arteries.

Another factor that may cause differences in the results of various studies is the study population sex distribution. So that in the Korean study, the increase in ISH prevalence after the age of 60 years was reported to be higher in women than men [9]. In the Canadian study, also, the mean of ISH values at a younger age was lower for women than for men but higher after 60 years of age [34]. Another study in Taiwan has shown that, at all ages, the prevalence of ISH in women is higher than in men and increases with advancing age in both sexes [14].

In the present study, in line with the above-mentioned studies, the ISH prevalence was approximately higher in women in all age groups. However, Given that the above-mentioned studies [9, 14, 34] have not reported a P-value or confidence interval for the ISH prevalence in both genders, the comparison is partly difficult.

The results of the current study indicated that obesity increases the odds of ISH by 3.73 times. This is in line with the results of previous studies in South Korea [31], China [35], Taiwan [36], and rural India [7, 28]. For example, in a national survey in Korea, the odds ratio of developing ISH in obese people was 3.16 times more than those with a normal weight [31]. Also, another study in India has shown that obesity increases the odds of ISH by 2.21 [7]. According to the available evidence, in Asian people, obesity has a greater effect on hypertension than in Western people. So that in Asian people, the effect of BMI of 25 kg/m^2^ on blood pressure is similar to the effect of BMI of 30 kg/m^2^ in Western people [28]. It should also be noted that, as shown in the present study, obesity and overweight (BMI = 28.87 kg/m^2^) are highly prevalent in Iranian adults and it is a neglected priority [37, 38].

According to the results of this study, other factors significantly associated with the prevalence of ISH were diabetes and WHR which is consistent with the previous studies [31, 39, 40]. The prevalence of hypertension in diabetes patients is most common in comparison to the general population. So that in a population-based study, the prevalence of hypertension has reported 24% in type 1 diabetic and 60.2% in type 2 diabetes patients [41–43]. The association of diabetes and WHR with ISH might be related to the sympathetic nervous system, as the clinical and experimental evidence has shown a significant dependency between obesity, hypertension, hyperinsulinemia, and diabetes through the sympathetic nervous system [39, 41, 44–46]. This dependency may be due to the activation of the renin–angiotensin–aldosterone (RAA) system in type 1 diabetes and insulin resistance or hyperinsulinemia in type 2 diabetes patients [41, 47, 48].

The present study had several limitations. First, this study had a cross-sectional design, therefore, the observed association between ISH and its risk factors cannot be considered as a causal association and needs further investigation in prospective studies. Second, some of the previous studies have reported a significant association between alcohol intake, salt intake, and physical activity with the prevalence of ISH [35]. However, in this study, we were not collecting these variables. Third, must keep in mind that some ISH cases may not be included in the definition of ISH due to receiving antihypertensive medications. This may lead to an underestimation of the ISH prevalence. Four, In the present study, blood pressure was measured in one arm and blood pressure diagnosis was based on a single office visit. While usually 2–3 office visits at 1–4-week intervals and blood pressure measurements in both arms are recommended to confirm the diagnosis of hypertension.

## Conclusions

The present study showed that isolated systolic hypertension was highly prevalent among the middle-aged and older adult population in Iran. Several risk factors including age, BMI, WHR, and diabetes were associated with ISH prevalence. Given the risk of cardiovascular disease associated with ISH, these results emphasize the importance of education and the need for public health interventions to improve the detection, prevention, and treatment of ISH.

## Data Availability

The datasets used and/or analyzed during the current study are available from the corresponding author on reasonable request.

## Abbreviations

ISH: Isolated Systolic Hypertension
ShECS: Shahroud Eye Cohort Study
WHR: Waist to Hip Ratio
OR: Odds Ratio
DALYs: Disability Adjusted Life Years
BP: Blood Pressure
IDH: Isolated Diastolic Hypertension
SDH: Systolic Diastolic Hypertension
FBS: Fasting Blood Surge
BMI: Body Mass Index
RAA: Renin Angiotensin Aldosterone
SD: Standard Deviation
CI: Confidence Interval
